# The Use of Epispastics

**Published:** 1856-12

**Authors:** Wm. Johnson

**Affiliations:** White House, N. J.


### ON THE OCCASIONAL ILL EFFECTS RESULTING FROM THE USE OF EPISPASTICS.

BY WM. JOHNSON, M. D., WHITE HOUSE, N. J.

Some of the most valued articles of the materia medica do occasionally act very adversely to the expectations of the prescriber; and, instead of producing salutary impressions, manifest destructive tendencies. The records of such therapeutic operations may prove as beacon lights to the junior members of the profession, leading them to caution in the use of medicines which, if they do not give a quietus to disease, may inflict deadly injuries upon the patients themselves. As illustrations ot these truths, I need merely advert to the ill effects occasionally resulting from mercury, antimony, hydrocyanic acid, digitalis and a host of other highly esteemed medicaments. Every physician of much experience can call up cases where he has been sadly disappointed in the results of his medication from idiosyncrasy and other fortuitous circumstances. I intend, however, to confine my remarks at this time to a single article of the materia medica, and one in almost universal use; namely, the Cantharis Vesicatoria, or Spanish Fly. For the natural history of this insect I refer to works on Entomology. All the information necessary on this subject may, however, be readily obtained by turning to the article *Cantharis*, in that truly national work, the *United States Dispensatory*, by Wood and Bache. I pass over all the interesting matter contained under that article, until I come to the ill effects occasionally resulting from the use of the cantharides. Dr. Wood says: "On some constitutions they produce a poisonous impression, attended with frequent pulse, dryness of the mouth and fauces, heat of skin, subsultus tendinum, and even convulsions; and some physicians have been so much alarmed by the occasional occurrence of these symptoms as to induce them to employ the remedy with great hesitation. What is the precise condition ot the system in which the effects result, it is impossible to determine."

I have prescribed the cantharides from the commencement of my practice. From their internal use I have never seen any unpleasant consequences arise, except a slight strangury, and I have given them in tincture in the large doses advised by Dr. Dewees in uterine affections. I have, on the contrary, seen the most pleasing effects produced, and I have prescribed the article to a very great extent. I cannot, however, bear the same testimony to their external use. In using the flies as an epispastic, I have been governed by Dr. Rush's notion of the *blistering point*, and have not applied my plasters either during exalted and unsubdued arterial excitement, or in those conditions of the system where the *vires vitæ* were greatly prostrated; and my blisters have not been left on as long as is generally advised. Yet I have met with many cases where I had reason to regret the application of the epispastic. I have seen death, I think, produced by them in several instances. The diseases in which they had been prescribed were removed, but the patients died, in my opinion, from the action of the epispastic. In three instances of pneumonia in children, the epispastic arrested the onward course of the disease, and the cases appeared progressing to a favorable issue, but the blistered surfaces became gangrenous, or deep corroding ulceration took place, after the lapse of a few days, and the little patients sank.

In a case of obstinate vomiting in a child resisting all medication, a small epispastic was applied to the epigastrium, and the disease removed; but the blistered surface refused to heal, became gangrenous, and the patient died. Blistering in remitting and intermitting fever, in the early part of my professional life, was more frequently resorted to, to interrupt the febrile movements, than is done at present, and I saw death, I think, in a youth, produced by such medication. The blistered surfaces became gangrenous.* I have in one or two instances seen gangrene produced by this cause where the case did not prove fatal, but yielded to remedial appliances. I witnessed another instance where an epispastic, applied to the abdomen in enteric fever, proved fatal to the patient after a tolerably fair prospect of recovery had taken place. These instances of fatal vesication did not all occur in my own practice, nor were the epispastics all advised by myself. But they all fell under my observation. One of these fatal cases occurred in the practice of a neighboring physician. It was a child of about eighteen months. I saw the patient seveıal times. The pneumonia for which the blister was applied was cured, but the blister destroyed the child. Another of these unfortunate cases occurred in a child of about one year old; it too was pneumonia. The mother, without consulting me, applied the epispastic. Deep corroding ulceration rendered this case likewise fatal.

I pass by strangury and some other unpleasant effects produced by the absorption of the cantharides, and notice another case which fell under my care a number of years since. It was that of a lady of a most heroic disposition. She would bear the extraction of several molar teeth at one sitting, without uttering a groan, and during an accouchment a person in the room whose back was turned towards her, would not be suspicious that the throes of labor had expelled her child. Yet this woman would faint away whenever a blister was dressed on her person. For some years before her death, I never would apply one to her.

In addition to the fatal cases which I have related, I may here mention another, where I strongly suspected death to have resulted from the action of the cantharides. An adult aged about 35 years had complained of pain in the head. An epispastic was opplied to the *nucha*, and the blistered surface became unusually sore. Much nervous disorder was produced, and the patient died very suddenly. What share the epispastic had in bringing about this result, I cannot pretend to say. Whether the pain for which the blister was applied was premonitory of the apoplexy

*I would not, however, speak too positively in this case. Death may have resulted from some other and occult cause. I will state the case, and leave the reader to draw his own conclusions. The blisters were applied on the forearms early in the second week of the fever, which was a bilious remittent. After the lapse of two or three days, an unaccountable collapse in the patient's system took place. The pulse lost its volume, and became wiry; the skin its warmth, and became cold and clammy; the blisters their florid appearance, and became dark and gangrenous, and the patient rapidly sank.

which produced sudden death (the death, however, was rather too sudden to be referred to this cause—apoplexy,) and which might have occurred without the intervention of the blister; or whether the blister itself produced a poisonous impression on the nervous system, I am unable to decide. I suspected strongly, however, the agency of the blister in this unfavorable result.

But I hasten on to the relation of another case which fell under my observation very lately, and which has elicited this communication. It is extracted from my Case Book, and is as follows:

June 9, 1856. W. A., farmer, aged about 30 years, black hair, dark skin, and of an hereditary predisposition to phthisis pulmonalis; has had several severe attacks of bronchitis. The irritation appears to be seated in the upper part of the trachea. His cough is almost incessant, but without expectoration. His pulse is about 80, tongue very slightly furred. In the medication employed, was an epispastic to the trachea and along and a little below the clavicles. This was applied day before yesterday, and was suffered to remain on about eight hours, when it was dressed with cabbage leaves. Nothing unusual was noticed until this morning. The blistered surface then became very painful, and a bread and milk poultice was applied. No relief, however, was afforded, and my son, Dr. Thomas Johnson, saw him. He directed the continuance of the poultice, having first sprinkled over the blistered surface, one-third of a grain of sulph. morphia, and directed it to be repeated every four or five hours, if the pain continued. He left him also the one-sixth of a grain of sulph. morph., to be repeated every three or four hours, if pain required it, and also forty drops ext. valerian, frequently repeated, to be taken internally. Very frequent spasms took place, however, during the night. These spasms are violently clonic.

10th. The patient is but little relieved; blistered surface very painful; frequent convulsive starts of the muscles of the arms and legs, which have kept him awake nearly all the past night—has not slept one hour during the night. My son directed dressing blister with pulverized slippery elm suspended in a strong decoction of hops; gave him one-sixth of a grain of morphia every four hours, with ext. valerian, forty drops, and lac. assafœtidæ, each dose containing five grains assafœtidæ. His cough is better.

11th. Patient is much better; pulse 60; tongue little furred; convulsive motions have entirely ceased; blister looks much better—the unfavorable aspect which it wore day before yesterday is now gone; patient has taken the valerian and lac. assafœtidæ alternately every four hours; has taken but one or two doses of the morphia since yesterday; he coughs again rather more than yesterday, but expectorates more; cauterized the air-passages again to-day.

I have now given the results of forty-five years' observation in a very large country practice. Perhaps I have had more of these unfortunate cases than fall to the lot of my professional brethren. In consulting Dr. Coxe and other writers on the Materia Medica, I find no allusion to the results which I have stated.*

The epispastics which I have employed were, in the early part of my professional life, powdered cantharides, dusted thickly over a plaster of basilicon ointment; but for many years back, I have been in the habit of using the common blistering ointment spread on muslin. I seldom suffer the plaster to remain on more than six or eight hours, or until the skin becomes pretty intensely inflamed, when I remove it, and apply a bread and milk poultice. In children, I leave the plaster on no longer than to inflame the skin, and then apply the bread and milk poultice. I afterwards dress the blistered surfaces with a plaster of beeswax and hog's lard. This dressing is not so offensive as the old fashioned dressing of wilted cabbage leaves. In nearly all the fatal cases which I have seen from the application of epispastics, the cabbage leaves were used as a dressing. I thought it necessary to make these statements, to show that there was nothing in the management of these cases to account for the fatal results of some of them.

Now what are the lessons to be learned from these unfortunate results of vesicating applications? Obviously, caution in their use. Let the medical prescriber remember, in resorting to them, he applies a resource of our profession pregnant with weal or woe. A question here arises: are we, in view of such contingencies as may arise from vesication with cantharides, justifiable in resorting to such a remedy? Let me answer the question by another quotation from Prof. Wood, which closes the article from which I have before quoted. Speaking of the unpleasant effects occasionally produced by this vesicatory, he says: "In this respect the Spanish flies are analogous to mercury; and any argument, drawn from this source, against the use of the one, would equally apply to the other. The general good which results from their use far overbalances any partial and uncertain evil."

Objections apply, however, with greater force to cantharides than to mercury. Whilst I have never in my own practice seen a single death to result from the employment of mercury, antimony, or hydrocyanic acid (although I am in the constant

---

*Dr. Chapman, in his Therapeutics, states that he has seen *gangrene* produced in two or three instances, by leaving the blisters on *too long*. He advises their being left on eight or ten hours. In none of the fatal cases which I have related were they left on much over half that time.

habit of prescribing them all,) I have seen five, if not six deaths, where I had very strong suspicions that vesication with cantharides was the cause. Yet I still think that, in a vast majority of cases, they are more efficient than any other articles of the materia medica in salutary local impression, and in this way often subverting the unfavorable tendency of disease. In this respect, I believe that we have no other epispastic that can be compared with them. In view, however, of the dangers which may result from their use, does it not become us to look out substitutes which may answer our purposes as counter-irritants? Dr. Todd, one of the most distinguished metropolitan physicians of Great Britain, has taught us that pneumonia may be cured without blisters or the lancet. His practice, both private and hospital, has been attended with remarkable success. The remedy in which he most confides is stupes of warm spirits of turpentine, applied freely to the chest, and left on half an hour at a time, and used three times a day. His internal treatment consisted in large doses of acetate or citrate of ammonia, given every three hours. The doses employed were from six to eight drachms. An aperient was occasionally given, and opium sometimes administered with calomel, and a supporting diet of beef-tea. The reason, however, which induced Dr. Todd to give the preference to turpentine over cantharides, was that it causes less embarrassment to the employment of the stethoscope. Not having a raw surface to apply the instrument over, it can be used more frequently and satisfactorily, and the changes resulting from disease more easily determined. Now pneumonia is one of the most frequent diseases in which we resort to epispastics, and in children, I think that they should be dispensed with. I have for many years treated the pneumonia of children without them, and have not often regretted the omission. I have gotten along well with turpentine, croton oil, and sinapisms. Cases may, however, occur in which we are compelled, as it were, to their use; let them then be employed intelligently, and with a full estimate of the risks to be encountered in their application.—*Med. & Surg. Reporter.*

